# Supplementary figures and images for: Drivers of respiratory syncytial virus seasonal epidemics in children under 5 years in Kilifi, coastal Kenya
Source: PLoS One. 2022 Nov 28;17(11):e0278066. doi: 10.1371/journal.pone.0278066 (PMC9704647; doi:10.1371/journal.pone.0278066)

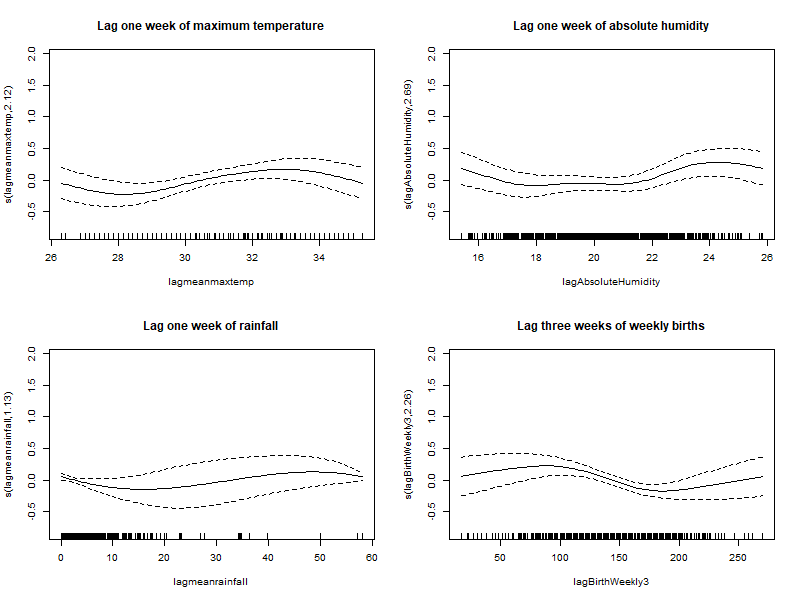

Supplement: S1 Fig — The dashed lines are the 95% confidence intervals. (TIF) [file pone.0278066.s002.tif]

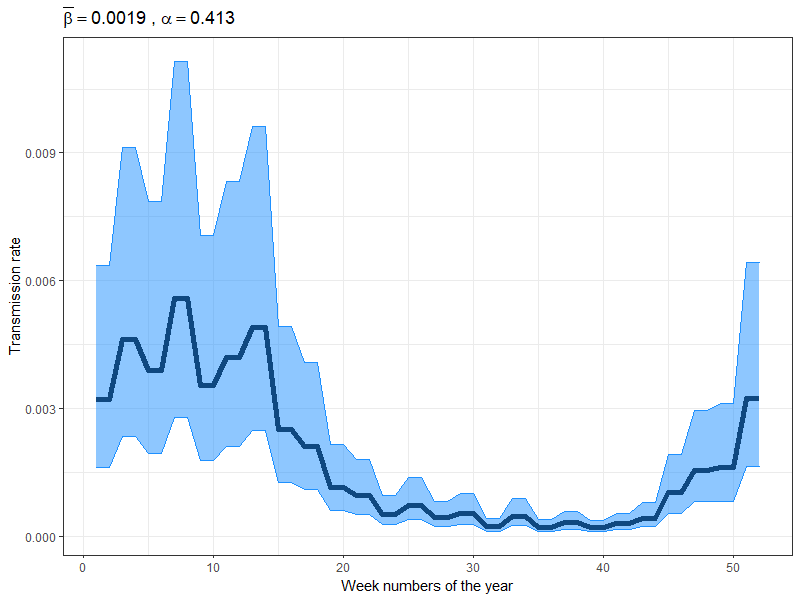

Supplement: S2 Fig — (TIF) [file pone.0278066.s003.tif]

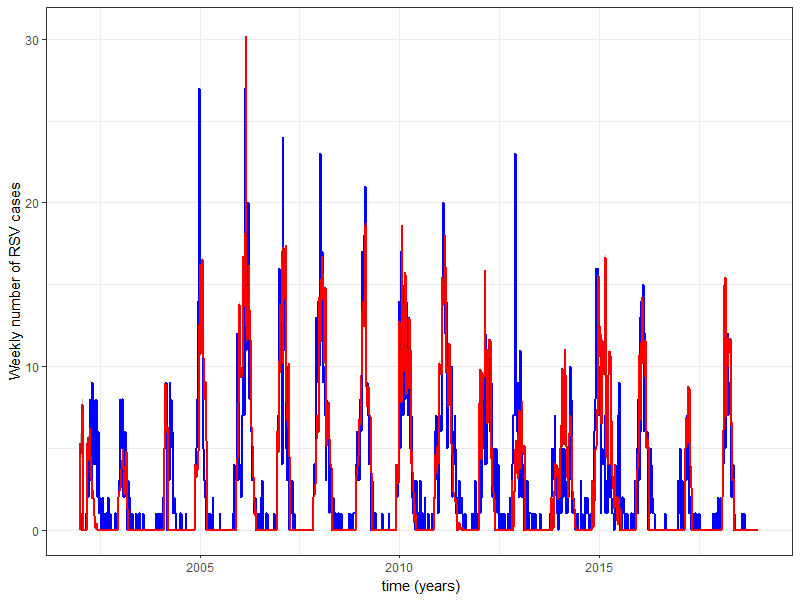

Supplement: S3 Fig — (TIF) [file pone.0278066.s004.tif]

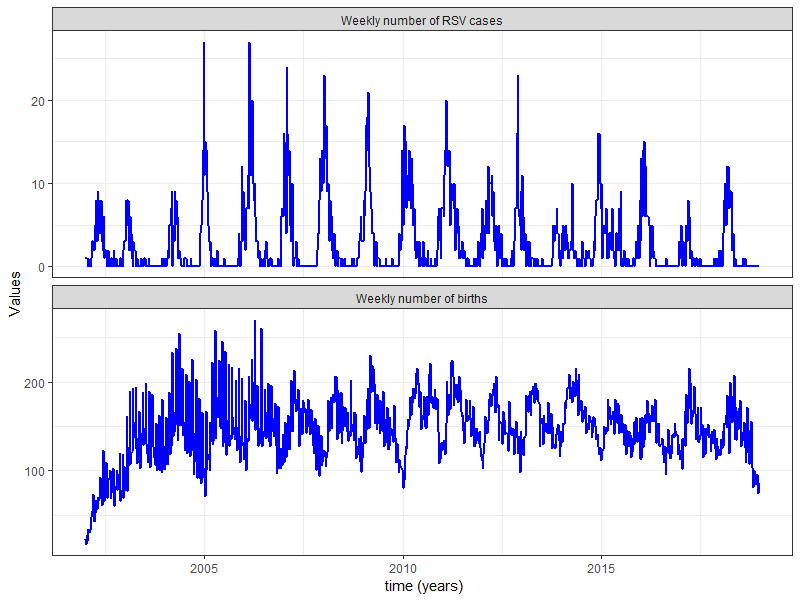

Supplement: S4 Fig — (TIF) [file pone.0278066.s005.tif]

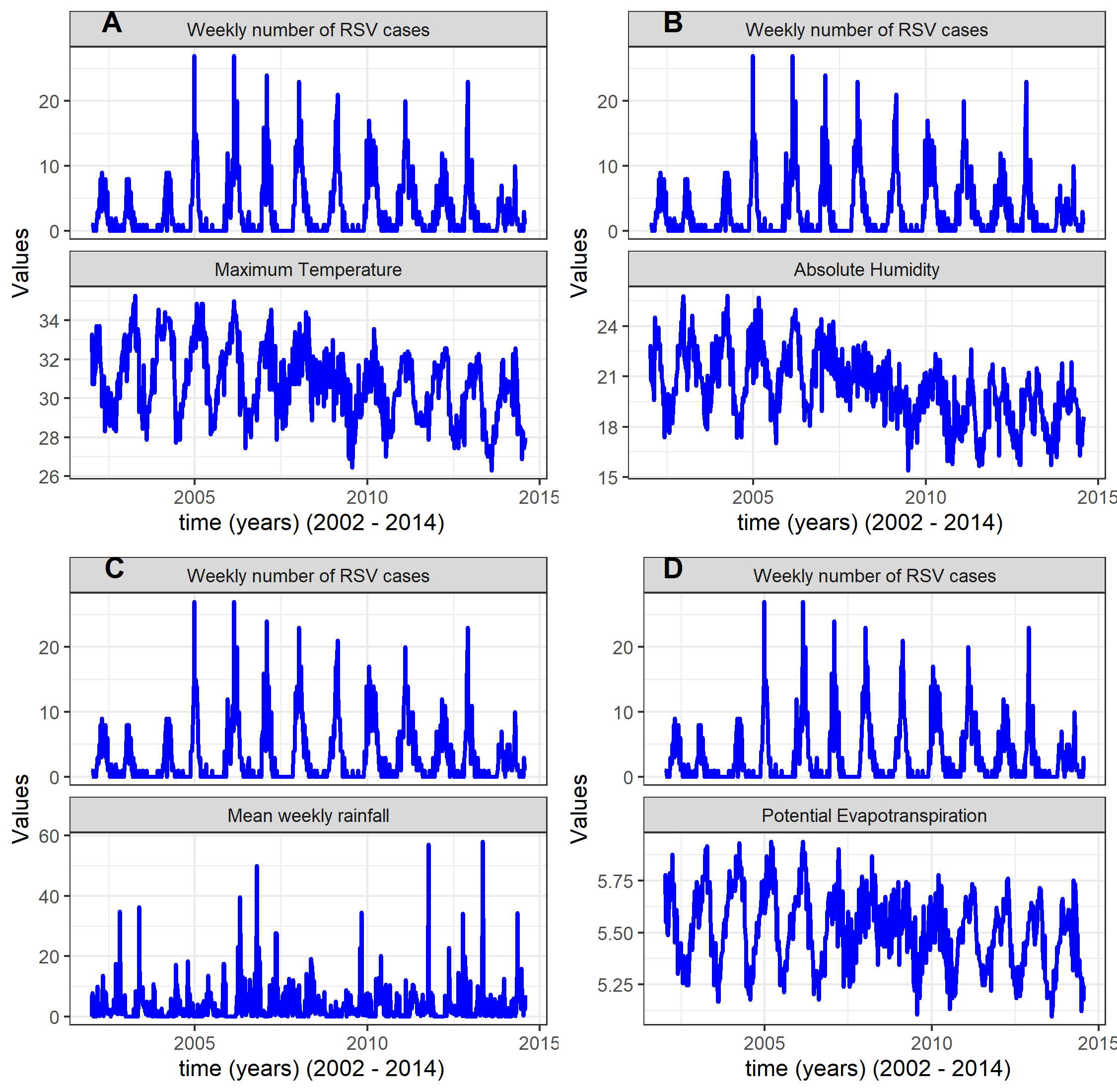

Supplement: S5 Fig — (TIF) [file pone.0278066.s006.tif]

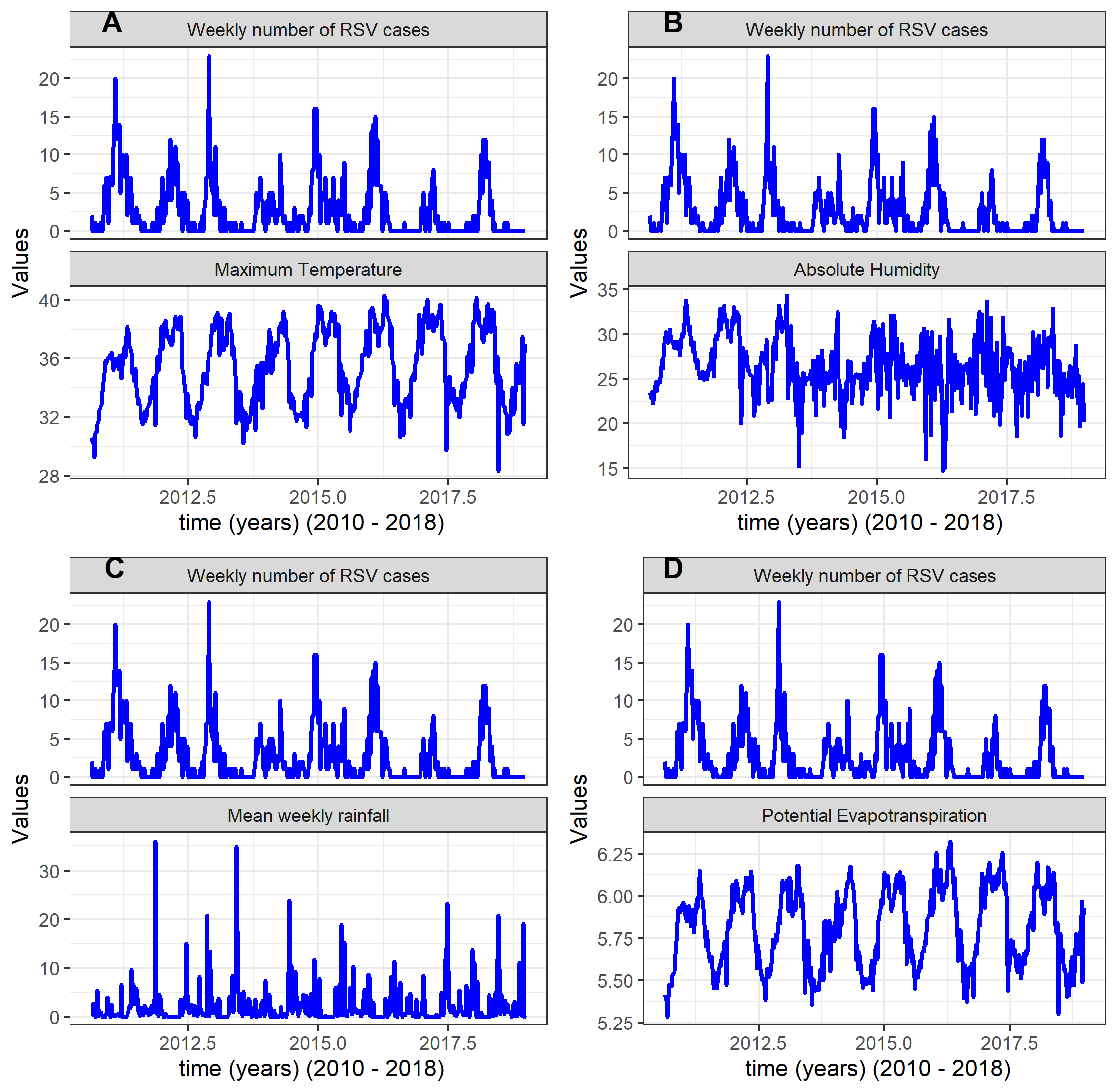

Supplement: S6 Fig — (TIF) [file pone.0278066.s007.tif]
