# Supplementary material for: Drivers of respiratory syncytial virus seasonal epidemics in children under 5 years in Kilifi, coastal Kenya
Source: PLoS One. 2022 Nov 28;17(11):e0278066. doi: 10.1371/journal.pone.0278066 (PMC9704647; doi:10.1371/journal.pone.0278066)
Supplement: S1 File — (DOCX) [file pone.0278066.s008.docx]

Drivers of respiratory syncytial virus seasonal epidemics in children under 5 years in Kilifi, coastal Kenya

James Wambua, Patrick K Munywoki, Pietro Coletti, Bryan Nyawanda, Nickson Murunga,

D. James Nokes, Niel Hens

**Supplementary Information**

**Data pre-processing**

Absolute humidity was obtained using (equation 1), where **T** is the average weekly temperature, **RH** is the average relative humidity, and **e** is the natural logarithm [1].

$\mathrm{AH} (\mathrm{grams}$/m^3^$)=\frac{6.112 x e[17.67 x T / T + 243.5] x RH x 18.02}{(273.15 + T) x 100 x 0.08314}$ (1)

Absolute humidity is a measure of the amount of water vapor in a unit volume of air, while relative humidity is a ratio of the amount of water vapor in a given temperature relative to the maximum amount the air can hold at that temperature [2]. We performed parallel analyses for the 2 meteorological datasets. The main unit of analysis was one week. Thus, of interest, were the weekly averages of these meteorological variables. Due to some missing data in the 2 meteorological datasets, multiple imputation using multivariate imputation by chain equations (MICE package) [3] implemented in the R Statistical software was performed.

In the dataset 2002 - 2014, percentages of the missing data were as follows; maximum temperature (8.2%), minimum temperature (8.2%), relative humidity (7.8%), and rainfall (3.4%). While percentages of missing data for the dataset 2010 - 2018 were as follows; maximum temperature (3.6%), minimum temperature (3.6%), rainfall (3.6%), and relative humidity (4.9%). To decide whether these two datasets could be merged, we performed an exploratory analysis of the data within the overlapping period 2010 - 2014. Daily values of maximum and minimum temperature from the two datasets were used. Our main aim was to explore and establish whether the datasets were different. We divided the maximum temperature values with the numerator values being data from the automatic weather station (AWS). The mean value was 1.170, the median value was 1.172, and the interquartile range (IQR) equalled 1.130 - 1.213. The results from the minimum temperature were, mean: 1.017, median: 1.017, and IQR 0.997 - 1.039. We obtained a time series plot, with y-axis as the resulting values and x-axis as dates falling within the overlapping period. The curve plot for the maximum temperature was consistently higher than the curve for the minimum temperature. Based on this, we decided to perform parallel analyses for both datasets as attempts to decide on an optimal multiplicative value could result in initiating errors in our analysis consequently biasing the study findings.

These datasets were imputed using multivariate imputation by chained equations (MICE package) implemented in the R statistical software as above-mentioned. We performed multiple imputation of 5 datasets for each meteorological dataset resulting in 5 datasets for each meteorological dataset, so 10 datasets in total. The number of imputations (5) was chosen based on imputation guidelines by Rubin [4] on the percentages of missing data. The resulting datasets were merged with the RSV data, birth data, and data on school term by year and calendar week. We performed parallel analyses for the 2 meteorological datasets.

**Supplement information on Generalized additive models (GAM)**

We employed the backfitting algorithm as implemented in the ‘mgcv’ package in R [5]. This algorithm estimates each smooth component of additive model by iteratively smoothing partial residuals with respect to the covariates that the smooth relates to. The models were compared using both the Akaike Information Criterion (AIC) and the Generalized Cross Validation (GCV) scores [6]. Given the inherent problem of over-fitting in the generalized additive models, we set the value of gamma parameter to be 1.5. This parameter allows one to increase the penalty on each model’s degrees of freedom in order to increasingly produce more smooth models, as over-fitting is controlled by the degree of penalization [7]. We then inspected the plots of the residual model deviances to see whether there were autocorrelations in the residuals which could indicate possible presence of over-fitting in the models.

**Time Series Susceptible-Infected-Recovered (TSIR) Methodology**

To explore the plausible drivers of RSV seasonal epidemics, we applied two approaches. The first involved using a generalized additive negative binomial model, whilst the second entailed using the time-series-susceptible-infected-recovered model (TSIR) [8-10]. This discrete time epidemic model comprises 2 state variables, namely: the infected and the susceptible. Since the susceptible is unobserved, we reconstruct it using a set of recursive difference equations. Next, using the reconstructed susceptible population over time, we reconstruct an empirical transmission rate which is subsequently used as a dependent variable in both linear regression and generalized additive negative binomial models with meteorological variables and school term data as independent variables. We explore the relationship/associations up to a lag of one week of the independent variables.

As aforementioned, the TSIR model reconstructs the susceptible population through a set of difference equations as follows:

$\boldsymbol{S}_{\boldsymbol{t+1}}\boldsymbol{=}\boldsymbol{S}_{\boldsymbol{t}}\boldsymbol{+}\boldsymbol{B}_{\boldsymbol{t}}\boldsymbol{+ I}_{\boldsymbol{t}}\boldsymbol{+}\boldsymbol{\epsilon}_{\boldsymbol{t}}$ **(2)**

Where $\boldsymbol{I}_{\boldsymbol{t}}$ and $\boldsymbol{S}_{\boldsymbol{t}}$ represent the number of infected and susceptible individuals respectively, and **t** is the time period, with the generation time for RSV taken as 1 week. $\boldsymbol{B}_{\boldsymbol{t}}$is the number of

weekly births and $\boldsymbol{\epsilon}_{\boldsymbol{t}}$is additive noise, with **E[**$\boldsymbol{\epsilon}_{\boldsymbol{t}}$**] = 0**. To reconstruct the susceptible population at each time step, the $\boldsymbol{S}_{\boldsymbol{t}}$can be written as $\boldsymbol{S}_{\boldsymbol{t}}\boldsymbol{=}$ ***Ŝ +*** $\boldsymbol{Z}_{\boldsymbol{t}}$*,* with ***Ŝ*** the average number of susceptible individuals in the population and $\boldsymbol{Z}_{\boldsymbol{t}}$ represents the unknown deviation from the average number of the susceptible individuals at each considered time step. We proceed by rewriting equation (2) in terms of deviations of $\boldsymbol{Z}_{\boldsymbol{t}}$and iterate successively starting at $\boldsymbol{Z}_{\boldsymbol{0}}$: yielding the following;

$\sum_{\boldsymbol{i=0}}^{\boldsymbol{t-1}} \boldsymbol{Bi}\boldsymbol{=}\boldsymbol{Z}_{\boldsymbol{0}}\boldsymbol{+ 1/\rho}\sum_{\boldsymbol{i=0}}^{\boldsymbol{t-1}} \boldsymbol{Iri}\boldsymbol{+}\boldsymbol{Z}_{\boldsymbol{t}}\boldsymbol{+}\boldsymbol{\epsilon}_{\boldsymbol{t}}$ **(3)**

Where $\boldsymbol{\rho}$represents the reporting rate accounting for both RSV infections/episodes that were not hospitalized as well as under-reporting of RSV hospitalizations. ***Iri*** is the reported incidence of the number of cases. Using equation (3), $\boldsymbol{Z}_{\boldsymbol{t}}$ is then estimated as the resulting residuals from the linear regression of the cumulative births on cumulative cases, assuming $\boldsymbol{\epsilon}_{\boldsymbol{t}}$ is small. The $\boldsymbol{\rho}$is computed as the inverse of the slope of the regression model and $\boldsymbol{S}_{\boldsymbol{t}}$is constructed from the $\boldsymbol{Z}_{\boldsymbol{t}} \mathrm{and}$***Ŝ*** estimates which entails defining the expected number of infected cases at each time step **E[I_t+1_]** as:

$\boldsymbol{E[It+1]}\mathbf{=}\frac{\boldsymbol{\beta}\boldsymbol{t} {\boldsymbol{I}\boldsymbol{t}}^{\boldsymbol{\alpha}} \mathbf{S}\boldsymbol{t}}{\mathbf{N}\boldsymbol{t}}$ **(4)**

Which is log-linearized as:

***ln (E[I_t+1_])***$\boldsymbol{= ln (}\boldsymbol{\beta t}\boldsymbol{) +}$***α ln (I_t_) + ln (Ŝ +*** $\boldsymbol{Z}_{\boldsymbol{t}}\boldsymbol{)}$ ***– ln (***$\boldsymbol{Nt}$***)* (5)**

Where the $\boldsymbol{\beta t}$ are weekly factors that represent the changing transmission rate and the$\boldsymbol{\alpha}$ is a constant that captures the underlying heterogeneities in the mixing patterns as well as the discretization of a continuous time process into a discrete time framework. The population data ***Nt*** was obtained by performing a cubic spline interpolation using population surveillance data from the Kilifi Health and Demographic Surveillance System (KHDSS) [11]. The interpolation was done at weekly level assuming the population was monotonically increasing. We fit Equation (5) using the negative binomial due to overdispersion in the RSV cases. We then use Equation (5) to estimate the mean number of susceptible population, ***Ŝ***, using the marginal profile likelihood. We make an assumption same to Baker at al [8] that the first RSV infection is immunizing, as subsequent infections have reduced infectiousness and are less severe [12]. The $\boldsymbol{S}_{\boldsymbol{t}}$is then derived as $\boldsymbol{S}_{\boldsymbol{t}}\boldsymbol{=}$ ***Ŝ +*** $\boldsymbol{Z}_{\boldsymbol{t}}.$

The empirical estimate of the transmission rate, $\boldsymbol{Emp\beta t}$, is then computed in each time step by assuming that ***E[I_t+1_] = I_t+1_***

$$\boldsymbol{Emp}\boldsymbol{\beta t}\boldsymbol{=}\frac{\boldsymbol{I}\boldsymbol{t+1 *}\boldsymbol{N}\boldsymbol{t}}{{\boldsymbol{I}\boldsymbol{t}}^{\boldsymbol{\alpha}} \boldsymbol{S}_{\boldsymbol{t}}}$$

In order to avoid the inflation of the $, \boldsymbol{Emp\beta t},$we add one to the time series with zero values in order to avoid epidemic extinction as depicted by zero numbers in the data. We utilize the tsiR package [9] to fit the TSIR model and reconstruct the RSV seasonal epidemics which are then overlaid with the actual RSV cases. The $\boldsymbol{Emp\beta t}$is then used as a response variable in both a linear regression and generalized additive model where we assess the associations of meteorological variables (maximum temperature, absolute Humidity, rainfall) and school term.

**Results for the TSIR Methodology**

The predicted weekly RSV cases from the TSIR model showed a similar trend with the observed RSV cases (S3 Fig), indicating that weekly number of births could be a potential driver of RSV seasonal dynamics in this region. In the 2002-2014 datasets, results from the fitted linear regression model which utilized log transformed empirical transmission rates showed that maximum temperature was a significant predictor at both lag zero and lag one (p-value <0.05, S2 Table). Similarly, absolute humidity was a significant predictor of the log transformed empirical transmission rates at both lags zero and one (p-value <0.05, S2 Table).

The results from the fitted linear regression in the 2010-2018 datasets yielded similar results where maximum temperature was a significant predictor of the log transformed empirical transmission rates at both lags zero and one week (p-value <0.05, S3 Table). Absolute humidity yielded both significant and non-significant results (p-value <0.05, S3 Table).

Results from the generalized additive negative binomial models when the empirical transmission rates were utilized as the dependent variable indicated that maximum temperature and absolute humidity were significant predictors (p-value <0.05, S4 Table).

The estimated reporting rate from the TSIR model was 0.0217. This value is generally small

implying that either there were RSV hospitalizations which were not reported possibly due to

children being taken to different hospitals and thus not captured in the database or the RSV

infections did not result into hospitalizations perhaps due to mild levels of infections. The estimated transmission rate showed a seasonal pattern of RSV transmission which starts towards the last quarter of the year, peaks in the first quarter, and then declines again from the second quarter of the year (S2 Fig).

S1 Fig. Plots of the meteorological smooth terms in the first imputed 2002-2014 dataset. The dashed lines are the 95% confidence intervals.

S2 Fig. The estimated transmission rate from the time-series-susceptible-infected-recovered (TSIR) model with the 95% confidence interval.

S3 Fig. plot of the mean of 100 stochastic simulations from the TSIR model fit (red) plotted

alongside observed data of weekly RSV cases (blue).

S4 Fig. Plot for the weekly number of RSV cases and the weekly number of births from the year 2002 to 2018.

S5 Fig. Weekly number of RSV cases compared to mean weekly maximum temperature, absolute humidity, rainfall, and potential evapotranspiration between the year 2002 and 2014.

S6 Fig. Weekly number of RSV cases compared to mean weekly maximum temperature, absolute humidity, rainfall, and potential evapotranspiration between the year 2010-2018.

Table S1. Generalized additive negative binomial models with lags zero and one week of meteorological variables and lags two and three weeks of weekly number of births, respectively, in the 2010-2018 datasets.

|  | Smooth terms EDF (p-value) † | | | |  |  |  |  |
| --- | --- | --- | --- | --- | --- | --- | --- | --- |
| **Imputed**  **datasets** | **Maximum**  **Temperature**  **(Lag zero)** | **Absolute**  **Humidity**  **(Lag zero)** | **Weekly**  **Births**  **(Lag two)** | **Rainfall**  **(Lag**  **zero)** | **School**  **Term**  **(Lag zero)** | **Adj.R^2^** | **% Dev.**  **explained** | **Pred.**  **Corr. Coef**‡ |
| Data 1 | 6.217  (< 0.001) | 4.099  (0.022) | 7.134  (0.016) | 2.625  (0.300) | 0.004  (0.959) | 0.68 | 68.7 | 0.843 |
| Data 2 | 6.292  (< 0.001) | 2.597  (0.158) | 7.172  (0.007) | 7.723  (0.053) | 0.173  (0.039) | 0.679 | 69.2 | 0.845 |
| Data 3 | 5.914  (< 0.001) | 4.549  (0.001) | 7.434  (0.004) | 1.053  (0.065) | 0.118  (0.146) | 0.685 | 69.0 | 0.845 |
| Data 4 | 6.141  (< 0.001) | 4.980  (0.019) | 7.402  (0.004) | 0.217  (0.267) | 0.119  (0.138) | 0.686 | 68.6 | 0.827 |
| Data 5 | 6.165  (< 0.001) | 5.454  (0.004) | 7.336  (0.011) | 7.454  (0.144) | 0.111  (0.194) | 0.701 | 69.9 | 0.835 |
| Lag one of meteorological variables and school term | | | | | | | | |
| **Imputed**  **datasets** | **Maximum**  **Temperature**  **(Lag one)** | **Absolute**  **Humidity**  **(Lag one)** | **Weekly**  **Births**  **(Lag three)** | **Rainfall**  **(Lag**  **one)** | **School**  **Term**  **(Lag one)** | **Adj.R^2^** | **% Dev.**  **explained** | **Pred.**  **Corr. Coef**‡ |
| Data 1 | 6.808  (<0.001) | 3.438  (<0.001) | 6.556  (0.020) | 5.645  (0.153) | -0.068  (0.461) | 0.691 | 69.1 | 0.850 |
| Data 2 | 3.490  (<0.001) | 6.616  (<0.001) | 3.604  (0.010) | 7.714  (0.028) | 0.234  (0.007) | 0.691 | 69.0 | 0.850 |
| Data 3 | 4.165  (< 0.001) | 2.184  (0.003) | 3.459  (0.025) | 7.679  (0.061) | 0.251  (0.002) | 0.688 | 68.3 | 0.847 |
| Data 4 | 5.306  (<0.001) | 2.039  (0.020) | 2.55  (0.034) | 0.619  (0.101) | 0.213  (0.008) | 0.67 | 67.4 | 0.834 |
| Data 5 | 3.783  (< 0.001) | 6.109  (<0.001) | 3.400  (0.032) | 6.186  (0.262) | 0.185  (0.026) | 0.692 | 68.8 | 0.849 |

†EDF: Effective degrees of freedom for the estimated smooth terms.

‡ Correlation coefficient between the estimated and observed RSV

S2 Table. Linear models for the log transformed empirical transmission rates with lags zero and one week of the covariates in the 2002-2014 datasets.

| **Imputed**  **datasets** | **Maximum Temperature**  **(Lag zero)** | **Absolute Humidity**  **(Lag zero)** | **Rainfall**  **(Lag zero)** | **School Term**  **(Lag zero)** |
| --- | --- | --- | --- | --- |
| Data 1 | -0.0866  (< 0.001) | 0.0292  (0.0298) | 0.0034 (0.2460) | 0.0586  (0.1689) |
| Data 2 | -0.0874  (< 0.001) | 0.0306  (0.0228) | 0.0017 (0.5262) | 0.0578  (0.1761) |
| Data 3 | -0.0908  (< 0.001) | 0.0309  (0.021) | 0.002  (0.393) | 0.054  (0.204) |
| Data 4 | -0.0856  (< 0.001) | 0.0279  (0.036) | 0.003  (0.200) | 0.0534  (0.209) |
| Data 5 | -0.0908  (< 0.001) | 0.0296  (0.028) | 0.001  (0.527) | 0.059  (0.164) |
| Lag one of meteorological variables and school term | | | | |
| **Imputed**  **datasets** | **Maximum Temperature**  **(Lag one)** | **Absolute Humidity**  **(Lag one)** | **Rainfall**  **(Lag one)** | **School Term**  **(Lag one)** |
| Data 1 | -0.0760  (< 0.001) | 0.0280  (0.042) | 0.004  (0.1299) | 0.0667  (0.1199) |
| Data 2 | -0.0727  (< 0.001) | 0.0267  (0.0483) | 0.003  (0.170) | 0.065  (0.1264) |
| Data 3 | -0.0800  (< 0.001) | 0.0315  (0.020) | 0.003  (0.201) | 0.0627  (0.144) |
| Data 4 | -0.0738  (< 0.001) | 0.027  (0.0430) | 0.004  (0.123) | 0.065  (0.146) |
| Data 5 | -0.0793  (< 0.001) | 0.027  (0.0411) | 0.002  (0.349) | 0.067  (0.1170) |

S3 Table. Linear models for the log transformed empirical transmission rates with lags zero and one week of covariates in the 2010-2018 datasets.

| **Imputed**  **datasets** | **Maximum Temperature**  **(Lag zero)** | **Absolute Humidity**  **(Lag zero)** | **Rainfall**  **(Lag zero)** | **School Term**  **(Lag zero)** |
| --- | --- | --- | --- | --- |
| Data 1 | -0.0228  (0.0110) | -0.0203  (0.0021) | 0.0039  (0.438) | -0.034  (0.508) |
| Data 2 | -0.0335  (< 0.001) | -0.0158  (0.0143) | 0.008  (0.8608) | -0.1029  (0.036) |
| Data 3 | -0.0391  (< 0.001) | -0.0043  (0.539) | 0.0003  (0.938) | -1.1030  (0.0374) |
| Data 4 | -0.0370  (< 0.001) | -0.0063  (0.3491) | 0.0009  (0.846) | -0.1042  (0.035) |
| Data 5 | -0.0356  (< 0.001) | -0.0108  (0.099) | 0.0015  (0.764) | -0.106  (0.0301) |
| Lag one of meteorological variables and school term | | | | |
| **Imputed**  **datasets** | **Maximum Temperature**  **(Lag one)** | **Absolute Humidity**  **(Lag one)** | **Rainfall**  **(Lag one)** | **School Term**  **(Lag one)** |
| Data 1 | -0.0131  (0.149) | -0.019  (0.003) | 0.005  (0.275) | -0.0394  (0.449) |
| Data 2 | -0.0253  (0.0067) | -0.015  (0.022) | 0.0018  (0.711) | -0.084  (0.0903) |
| Data 3 | -0.0286  (0.004) | -0.0072  (0.3200) | 0.014  (0.780) | -0.082  (0.098) |
| Data 4 | -0.029  (0.002) | -0.0067  (0.321) | 0.0017  (0.730) | -0.085  (0.089) |
| Data 5 | -0.0283  (0.003) | -0.0089  (0.186) | 0.0028  (0.587) | -0.087  (0.079) |

S4 Table. Generalized additive negative binomial models with lags zero and one week of the meteorological variables and school term when the empirical transmission rate is used as the dependent variable in the 2002-2014 imputed datasets.

|  | Smooth terms EDF (p-value) † | | |  |  |  |
| --- | --- | --- | --- | --- | --- | --- |
| **Imputed**  **datasets** | **Maximum**  **Temperature**  **(Lag zero)** | **Absolute**  **Humidity**  **(Lag zero)** | **Rainfall**  **(Lag**  **zero)** | **School**  **Term**  **(Lag zero)** | **Adj.R^2^** | **% Dev.**  **explained** |
| Data 1 | 1.907  (0.015) | 2.846  (<0.001) | 1.394  (0.05) | 0.041  (0.147) | 0.584 | 60.7 |
| Data 2 | 1.813  (0.025) | 2.971  (<0.001) | 1.293  (0.069) | 0.042  (0.135) | 0.584 | 60.9 |
| Data 3 | 2.156  (0.006) | 2.902  (<0.001) | 0.817  (0.183) | 0.036  (0.194) | 0.586 | 61 |
| Data 4 | 2.179  (0.009) | 2.947  (<0.001) | 1.317  (0.062) | 0.039  (0.159) | 0.584 | 61.1 |
| Data 5 | 1.957  (0.014) | 3.045  (<0.001) | 1.208  (0.092) | 0.044  (0.113) | 0.585 | 61 |
| Lag one of meteorological variables and school term | | | | | | |
| **Imputed**  **datasets** | **Maximum**  **Temperature**  **(Lag one)** | **Absolute**  **Humidity**  **(Lag one)** | **Rainfall**  **(Lag**  **one)** | **School**  **Term**  **(Lag one)** | **Adj.R^2^** | **% Dev.**  **explained** |
| Data 1 | 2.172  (0.008) | 2.575  (<0.001) | 1.105  (0.078) | 0.034  (0.223) | 0.588 | 60.5 |
| Data 2 | 1.813  (0.035) | 2.707  (<0.001) | 0.865  (0.125) | 0.036  (0.202) | 0.588 | 60.5 |
| Data 3 | 2.412  (0.004) | 2.448  (<0.001) | 0.751  (0.157) | 0.031  (0.281) | 0.586 | 60.4 |
| Data 4 | 2.372  (0.010) | 2.529  (<0.001) | 0.950  (0.125) | 0.034  (0.236) | 0.586 | 60.3 |
| Data 5 | 2.449  (0.008) | 2.627  (<0.001) | 0.778  (0.171) | 0.036  (0.201) | 0.588 | 60.6 |

†EDF: Effective degrees of freedom for the estimated smooth terms.

**References**

1. *How to convert relative humidity to absolute humidity. .* 2012.

2. Soebiyanto, R.P., et al., *Associations between Meteorological Parameters and Influenza Activity in Berlin (Germany), Ljubljana (Slovenia), Castile and León (Spain) and Israeli Districts.* PLOS ONE, 2015. **10**(8): p. e0134701.

3. Buuren, S.v. and K. Groothuis-Oudshoorn, *mice: Multivariate imputation by chained equations in R.* Journal of statistical software, 2010: p. 1-68.

4. Rubin, D.B. *An overview of multiple imputation*. in *Proceedings of the survey research methods section of the American statistical association*. 1988. Citeseer.

5. Wood, S. and M.S. Wood, *Package ‘mgcv’.* R package version, 2015. **1**(29): p. 729.

6. Wood, S.N., *Generalized additive models: an introduction with R*. 2006: chapman and hall/CRC.

7. Kim, Y.J. and C. Gu, *Smoothing spline Gaussian regression: more scalable computation via efficient approximation.* Journal of the Royal Statistical Society: Series B (Statistical Methodology), 2004. **66**(2): p. 337-356.

8. Baker, R.E., et al., *Epidemic dynamics of respiratory syncytial virus in current and future climates.* Nature communications, 2019. **10**(1): p. 1-8.

9. Becker, A.D. and B.T. Grenfell, *tsiR: An R package for time-series Susceptible-Infected-Recovered models of epidemics.* PloS one, 2017. **12**(9): p. e0185528.

10. Finkenstädt, B.F. and B.T. Grenfell, *Time series modelling of childhood diseases: a dynamical systems approach.* Journal of the Royal Statistical Society: Series C (Applied Statistics), 2000. **49**(2): p. 187-205.

11. Scott, J.A., et al., *Profile: The Kilifi Health and Demographic Surveillance System (KHDSS).* Int J Epidemiol, 2012. **41**(3): p. 650-7.

12. White, L.J., et al., *The transmission dynamics of groups A and B human respiratory syncytial virus (hRSV) in England & Wales and Finland: seasonality and cross-protection.* Epidemiol Infect, 2005. **133**(2): p. 279-89.
