# Supplementary material for: Drivers of respiratory syncytial virus seasonal epidemics in children under 5 years in Kilifi, coastal Kenya
Source: PLoS One. 2022 Nov 28;17(11):e0278066. doi: 10.1371/journal.pone.0278066 (PMC9704647; doi:10.1371/journal.pone.0278066)
Supplement: Attachment — Submitted filename: Response to Reviewers.docx [file pone.0278066.s009.docx]

**Point-by-point reply for the manuscript with submission ID [PONE-D-22-07030] - [EMID:ea2b9abd7eba72cd]**

*Thank you for the opportunity to revise our manuscript entitled “*Drivers of respiratory syncytial virus seasonal epidemics in children under 5 years in Kilifi, coastal Kenya*”. We feel that we have adequately addressed all issues raised by the reviewers and we hope you are willing to consider our revised manuscript for publication in Plos One.*

*Hereunder, we have provided a detailed point-by-point reply to all comments raised by the reviewers together with a description of the changes we made in the revised manuscript (using red to denote the added information in the annotated version of the manuscript). The citations in this point by point response follow an independent enumeration with respect to the main manuscript.*

**Reviewer 1 Comments:**

**Thank you for submitting this valuable manuscript because it is the one of the most important topics to improve survival of children particularly in Sub-Saharan Africa. My comments and questions are as follows:**

We thank the reviewer for the appreciation of our work and for taking their time to review it and for their thoughtful comments and suggestions.

**Comments:**

1. **Introduction: In your introduction part, you stated a statement "Two maternal booster F protein vaccines are in Phase III trials", please site a reference.**

Thank you for pointing out this missing reference in our work. We have included a reference as suggested. The citation is entitled “Gunatilaka A, Giles ML. Maternal RSV vaccine development. Where to from here?. Human Vaccines & Immunotherapeutics. 2021 Nov 2;17(11):4542-8.”

2. **Methods: kindly give the study design even though you have used secondary data.**

This is an interesting suggestion. We have added a statement in the methods that indicate the study design for the pre-existing data. The statement reads, “*Data used were collected from routine longitudinal hospital case surveillance for RSV, and routine longitudinal surveillance for the number of births and meteorological variables*.”

**which GAM fitting method did you used? Backfilling algorithm or else?? please give some explanation about it.**

We used the local scoring algorithm, which iteratively fits weighted additive models by the backfitting algorithm as implemented in the mgcv package in R (1). The algorithm estimates each smooth component of an additive model by iteratively smoothing partial residuals, with respect to the covariates that the smooth relates to. The basic idea is that the partial residuals relating to the j^th^	smooth term are the residuals resulting from subtracting all the current model term estimates from the response variable, except the estimate of the j^th^	smooth term. This algorithm has the advantage of flexibility since it can choose from a wide variety of smoothing methods for component estimation (2).

**in your methods please give a description about model comparison methods used**.

We used both the Akaike Information Criterion (AIC) and Generalized Cross Validation (GCV) scores to compare different models.

**As you know overfitting is one problem that can be encountered in GAM. So, how do you assess the overfitting issue?? Kindly give explanation about it.**

We thank the reviewer for pointing this out. Indeed overfitting is one of the key problems that can be encountered when using the generalized additive models (GAM). Since the GAMs are simply generalized linear models estimated subject to smoothing penalties of the covariates, the most substantial difficulty is choosing the degree of penalization for the smoothing parameters which to a greater extend controls the overfitting. In our study, we used the package “mgcv” in R as indicated in the statistical analysis section and applied the Generalized Cross Validation (GCV) smoothing parameter estimation. Furthermore, we choose a value of 1.5 for the gamma parameter. This parameter allows one to increase the penalty on each models degrees of freedom in order to increasingly produce more smooth models. It has been demonstrated that a gamma of around 1.4 can be a sensible choice for suppressing over-fitting without much degradation in prediction error performance (3).

To assess whether there was presence of overfitting, we generated plots of the residual model deviances and investigated whether there was autocorrelation in these residuals.

Since we used two sets of time series datasets, i.e, 2002-2014 and 2010-2018, and considered five imputed datasets for each dataset, these datasets also acted as validation sets to each other. Fitted models in the imputed datasets yielded similar results and comparable adjusted R-Squared and the deviance explained further indicating no presence of over-fitting.

In order to provide information on these methodological clarifications in our work while keeping the statistical analysis section easy to follow, we created a section called “Supplementary information on Generalized Additive Models” in the Supplementary material and added the following information:

*We employed the backfitting algorithm as implemented in the ‘mgcv’ package in R. This algorithm estimates each smooth component of additive model by iteratively smoothing partial residuals with respect to the covariates that the smooth relates to. The models were compared using both the Akaike Information Criterion (AIC) and the Generalized Cross Validation (GCV) scores. Given the inherent problem of over-fitting in the generalized additive models, we set the value of gamma parameter to be 1.5. This parameter allows one to increase the penalty on each model’s degrees of freedom in order to increasingly produce more smooth models, as over-fitting is controlled by the degree of penalization. We then inspected the plots of the residual model deviances to see whether there were autocorrelations in the residuals which could indicate possible presence of over-fitting in the models.*

3. **Conclusion: you stated that school term is not significant predictor of seasonality of RSV, and you simply compare and contrast with findings of other previous studies please discuss the possible justification about it.**

This is an interesting point. The transmission dynamics of RSV in school aged children and the role of school settings are poorly understood. It remains uncertain as to whether the main driver of RSV transmission is primary infections or reinfections (4), if the former, and since practically all individuals experience a first infection by the third year of life (5), then school aged children would not be of importance. We conducted a surveillance study in a rural school in Kenya and identified very little RSV infection supporting the notion of little importance of RSV in the school setting (6). However, there are few studies on the epidemiology of RSV in school setting and further work in this area is warranted.

Based on the reviewer’s suggestion to provide a possible justification of why school terms don’t seem to play a crucial role, we add the following lines in the discussion section highlighting that more research needs to be done to shed light on the role of schools on RSV seasonality.

*Insights into why ‘school terms’ is not identified as a significant contributor to RSV seasonality would be gained from further studies of RSV transmission patterns in the school setting, including linking RSV individual-level infections data and social contact patterns for school going children at school-level, community and household levels to gain deeper understanding of ‘who infects who’ in the community.*

**Reviewer #2:**

1. **Tables: Table 1 is not captioned**

We thank the reviewer for pointing out this. The caption of the table is now included above the table (lines 231-233) and reads “*Table 1. Generalized additive negative binomial models with lags zero and one week of meteorological variables and lags two and three weeks of weekly number of births, respectively, in the 2002 - 2014 datasets*.”

2. **DISCUSSION: Be specific on maximum temperature, absolute humidity and weekly number of births. There should not be sub-headings under discussion**

We have now explicitly referred to maximum temperature and absolute humidity where this was not specific. We have also removed the sub-headings in the discussion section as per the reviewers suggestion.

3. **REFERENCES: In Vancouver reference style, it is only after 6 authors and their initials before ‘’et al’’ can be used (inserted)**

We have changed the reference style to Vancouver and now we have six authors and their initials before et al.

4. **CONTRIBUTION: The authors did not state the contribution of this study to the existing body of knowledge.**

This point is well noted. We have added the contribution of the study into the existing body of knowledge in the conclusion section and reads as follows: “*These findings contribute to the current understanding of the potential drivers of RSV seasonality in a tropical low-income setting where little is known about the plausible drivers of RSV seasonality*.”

5. **STUDY RESULTS IMPLICATION : Not stated**

We thank the reviewer for this comment. We have added the implication of the study results in the conclusion section and reads as follows. “*Furthermore, these factors can be utilized in the formulation of prediction models of RSV activity in this setting. This potentially could provide early warnings of RSV seasons and subsequently inform the timings of RSV immunizations using vaccine candidates currently in the pipeline*”.

**References**

1. Wood S, Wood MS. Package ‘mgcv’. R package version. 2015 Mar 31;1(29):729.
2. Wood SN. Generalized additive models: an introduction with R. chapman and hall/CRC; 2017, Second Edition.
3. Kim YJ, Gu C. Smoothing spline Gaussian regression: more scalable computation via efficient approximation. Journal of the Royal Statistical Society: Series B (Statistical Methodology). 2004 May;66(2):337-56.
4. Kinyanjui TM, House TA, Kiti MC, Cane PA, Nokes DJ, Medley GF. Vaccine induced herd immunity for control of respiratory syncytial virus disease in a low-income country setting. PloS one. 2015 Sep 21;10(9):e0138018.
5. Nyiro JU, Kombe IK, Sande CJ, Kipkoech J, Kiyuka PK, Onyango CO, Munywoki PK, Kinyanjui TM, Nokes DJ. Defining the vaccination window for respiratory syncytial virus (RSV) using age-seroprevalence data for children in Kilifi, Kenya. PLoS One. 2017 May 22;12(5):e0177803.
6. Adema IW, Kamau E, Nyiro JU, Otieno GP, Lewa C, Munywoki PK, Nokes DJ. Surveillance of respiratory viruses among children attending a primary school in rural coastal Kenya. Wellcome Open Research. 2020;5.

**Changes to the reference list**

We have added the following reference in the list

1. Gunatilaka A, Giles ML. Maternal RSV vaccine development. Where to from here?. Human Vaccines & Immunotherapeutics. 2021 Nov 2;17(11):4542-8.”
